# Hippocampal sharp-wave ripples in awake mice are entrained by respiration

**DOI:** 10.1038/s41598-017-09511-8

**Published:** 2017-08-21

**Authors:** Yu Liu, Samuel S. McAfee, Detlef H. Heck

**Affiliations:** Department of Anatomy and Neurobiology, University of Tennessee HSC, Memphis, TN 38163 USA

## Abstract

Several recent studies have shown that respiration modulates oscillatory neuronal activity in the neocortex and hippocampus on a cycle-by-cycle basis. It was suggested that this respiratory influence on neuronal activity affects cognitive functions, including memory. Sharp-wave ripples (SWRs) are high-frequency local field potential activity patterns characteristic for the hippocampus and implicated in memory consolidation and recall. Here we show that the timing of SWR events is modulated by the respiratory cycle, with a significantly increased probability of SWRs during the early expiration phase. This influence of respiration on SWR occurrence was eliminated when olfactory bulb activity was inhibited. Our findings represent a possible neuronal mechanism for a direct influence of the respiratory cycle on memory function.

## Introduction

In their 2006 opinion piece, Fontanini and Bower suggested that respiration-locked neuronal oscillations, which the olfactory bulb (OB) generates with almost every breath, might propagate throughout the entire neocortex via the olfactory system, creating a direct link between respiration and cortical neuronal rhythms^1^. In 2014 Ito *et al*. showed that indeed, OB respiration-locked oscillations were responsible for driving respiration-locked neuronal oscillations in the whisker barrel somatosensory cortex of the awake mouse^2^. The same study also showed that the power of neocortical gamma oscillations was modulated in phase with the respiratory cycle^2^. Because of the strong correlation of gamma oscillations with numerous cognitive functions including memory^3–7^, the phase-amplitude coupling between respiration and gamma power lead to speculations that respiration could directly influence cognitive functions associated with gamma oscillations^2, 8, 9^.

The hippocampus, a structure essential for memory function, is not formally a part of the olfactory system, but it receives inputs from the piriform cortex via the entorhinal cortex and exhibits respiration-locked oscillations^10–12^. The hippocampal network generates characteristic sharp-wave ripple (SWR) activity which has been shown to be critically involved in memory consolidation and memory retrieval in mice^13–15^, rats^16–23^ and non-human primates^24–26^.

In a recent study Zelano and colleagues showed that memory recall in humans is modulated by their respiratory phase, with subjects showing the most reliable memory recall of memorized items when they were presented during the inspiratory phase^27^. This effect occurred only when subjects were breathing through the nose, suggesting that respiration-locked OB activity was a necessary factor^27^. The neuronal mechanism behind the modulation of memory function with the phase of respiration is unknown.

Here we asked whether hippocampal SWR activity is modulated by respiration and whether respiratory influence on SWR activity required OB activation. To this end we performed extracellular recordings in the dorsal hippocampal CA1 region in awake head-fixed mice while simultaneously monitoring respiration. The role of olfactory bulb activity was evaluated using designer receptors exclusively activated by designer drugs (DREADDs)^28^ to inhibit respiration-locked activity in the OB.

## Results

**Figure 1:**
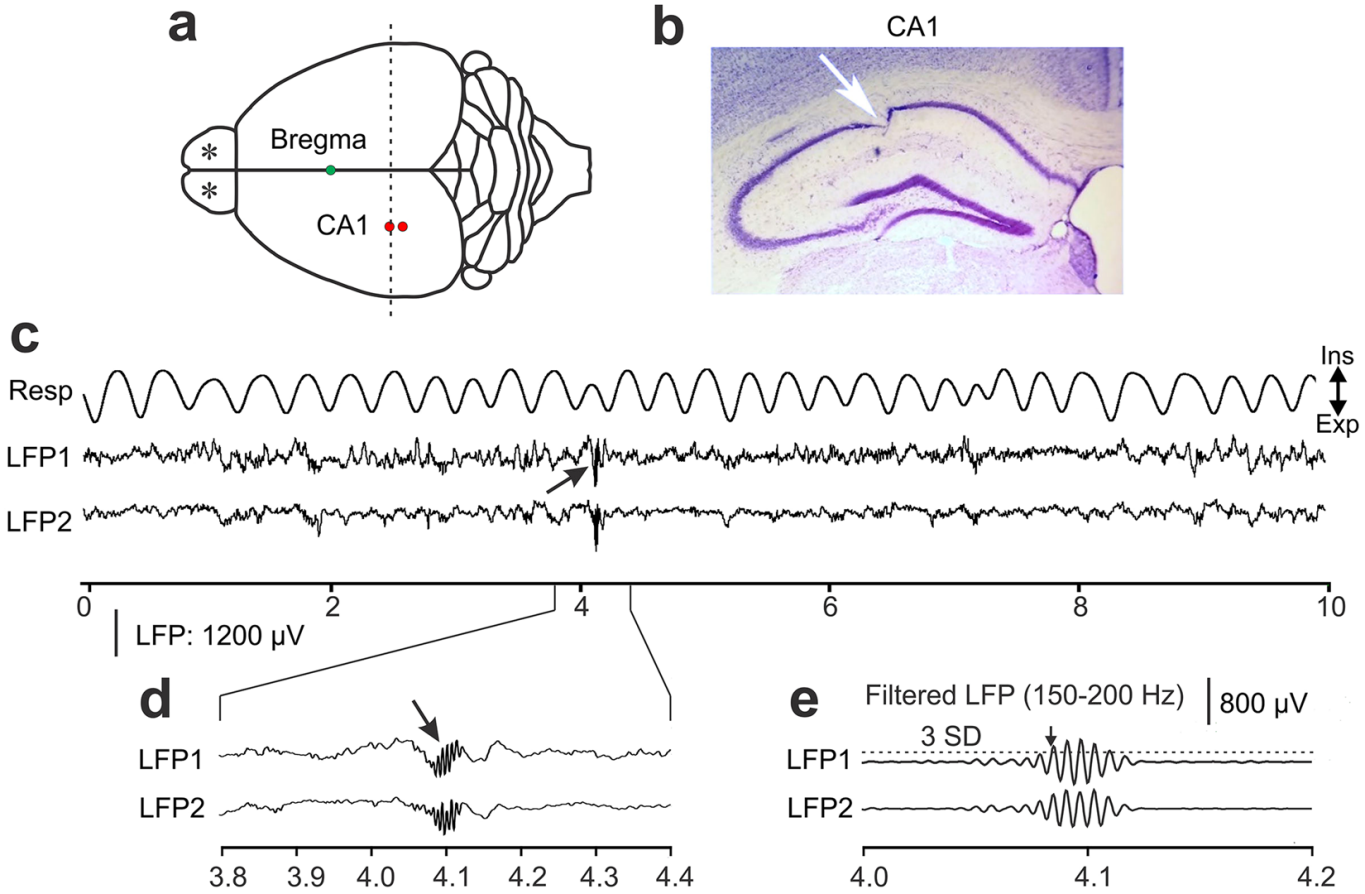
Recording sites and raw data examples of simultaneous recordings of local field potentials (LFPs) in the awake mouse. (**a**) Schematic drawing of the top view of a mouse brain. LFPs were recorded from the left CA1 region of the hippocampus. Dashed line represents the plane of the coronal section shown in (**b**) for verifying the recording location in CA1. Stars represent sites of bilateral injections of DREADD vector in OB. (**b**) An example of an electrolytic lesion (arrow) in a coronal section of the CA1 region of hippocampus. **(c)** Examples of respiration (Resp) and raw LFP data. The trace marked “Resp” shows respiration related temperature changes with expiration causing an increase in temperature, which corresponded to a decrease in voltage. The troughs in the trace thus represent the ends of expiration (Exp: expiration, Ins: inspiration). LFP1 and LFP2 were recorded from electrodes in CA1. Arrow points at a characteristic high-frequency ripple activity associated with SWRs. Abscissa represents time in seconds. (**d**) An enlarged view of raw LFPs around a hippocampal ripple event (arrow). Same LFP amplitude scale bar as in (**c**). (**e**) High-pass filtered versions of the LFPs in panel d emphasizes the high-frequency ripple components of CA1 activity. Horizontal dashed line above LFP1 represents the mean filtered LFP amplitude plus 3 standard deviations (SD) from a continuous recording of 60 seconds, which was used as a threshold (mean ± 3 SD) for automatic detection of sharp-wave ripple activity in the CA1 region. The arrow marks the beginning of ripple activity.

**Figure 2:**
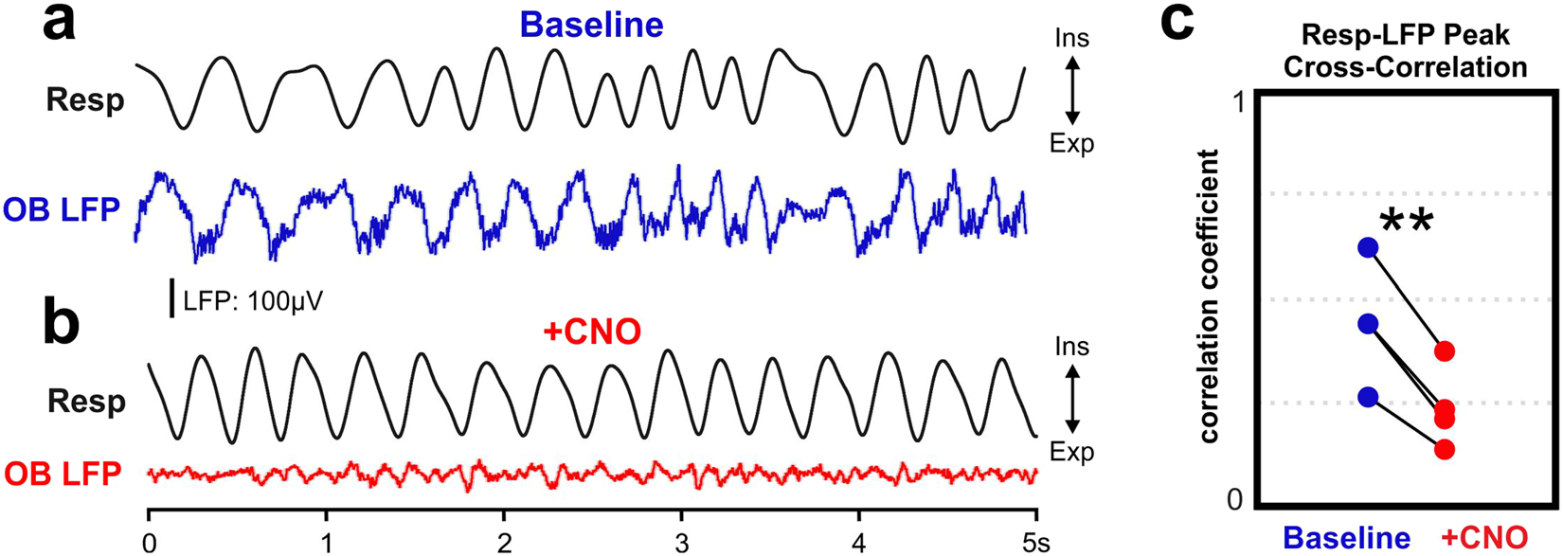
Disruption of olfactory bulb respiration-locked oscillations with administration of inhibitory DREADDs channel activator Clozapine-N-oxide (CNO). (**a**) Baseline example of simultaneously recorded olfactory bulb LFP and respiration shows robust neuronal oscillations in OB locked to the respiratory cycle. Time and voltage scale apply to (**a**,**b**). Troughs in respiration trace correspond to the end of expiration. (**b**) Example of olfactory bulb LFP after systemic injection of CNO solution. Respiration-locked neuronal oscillations are visibly disrupted. (**c**) Group data showing disruption of respiration-locked OB activity following CNO injection. Peak correlation values were taken to account for variable latency of respiration-driven oscillations. 10 minutes of resting data were used to calculate each cross correlation. Paired T-test: **p < 0.01. Exp: expiration; Ins: inspiration.

We used extracellular recording techniques in awake, head-fixed mice to measure local field potentials in the dorsal hippocampal CA1 region while simultaneously monitoring respiratory activity using a thermistor placed in front of the nose (Fig. 1a–c). The LFP signal was band-pass filtered at 150–200 Hz to facilitate the detection of SWR events using a threshold function (Fig. 1). Recordings of hippocampal SWR activity were conducted in nine mice. Five mice were assigned to a control group and 4 mice to a group that received bilateral injections of a viral vector construct that caused the expression of DREADDs in afferent projections to the main OB. This allowed us to temporarily suppress respiration-locked OB activity by systemic injection of CNO, as verified in separate experiments involving recordings of OB activity in an additional 4 mice (Fig. 2).

**Figure 3:**
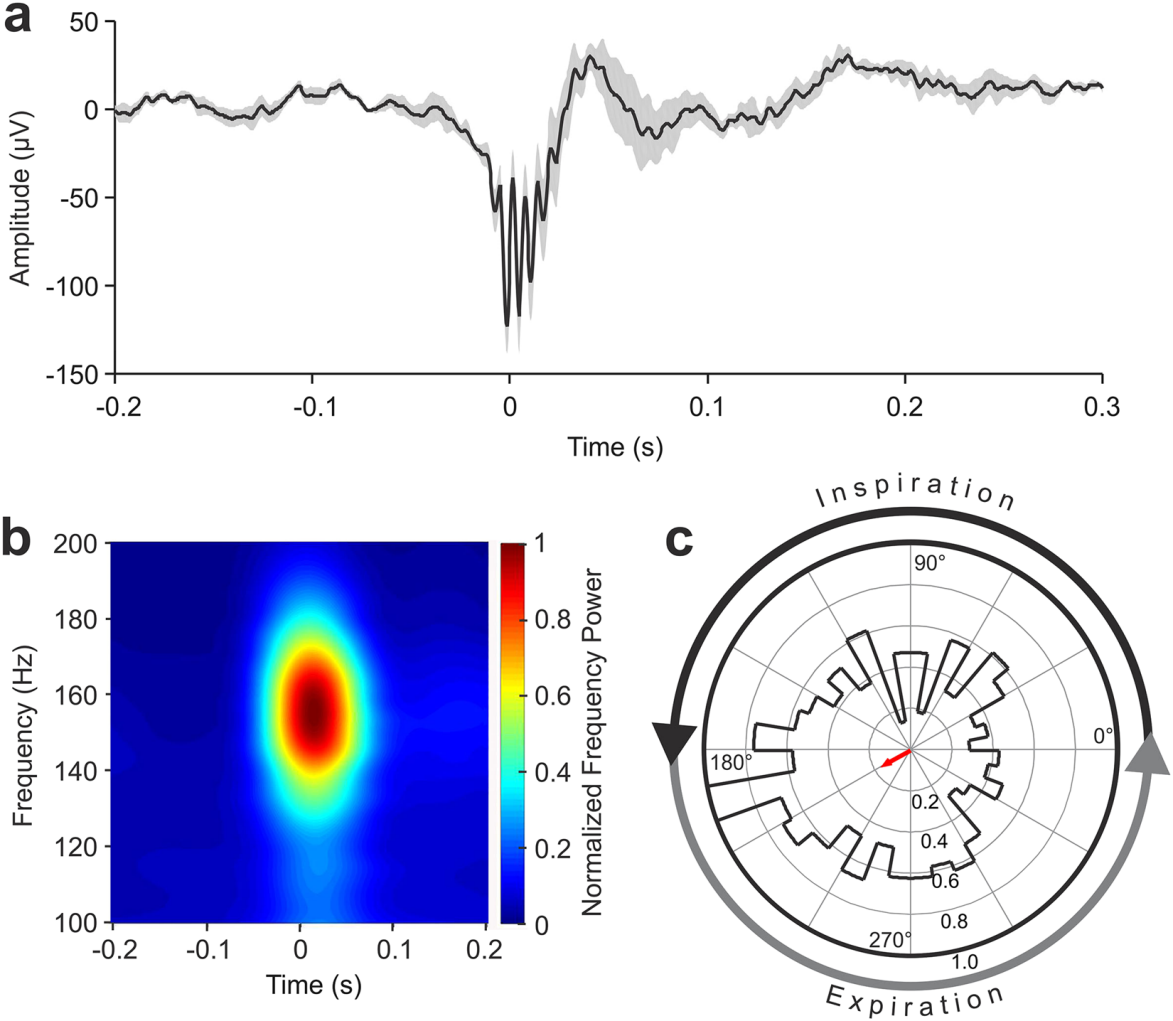
Hippocampal sharp-wave ripple (SWR) activity in relation to the respiratory cycle in mice. (**a**) Average local field potential (LFP) aligned on hippocampal SWRs (mean +/− standard error). Data are aligned on the onset of ripple-activity (at time 0 s). (**b**) Time-Frequency mapping of LFPs around CA1 ripples. Color represents normalized frequency power. (**c**) Polar plot reflecting the distribution of SWR events relative to respiratory phase. Red arrow represents the mean vector determined by circular statistics (Rayleigh test: n = 382; r = 0.14; z = 7.35; p = 0.02). 0° represents the end of expiration, 180° corresponds to the end of inspiration. Concentric circles mark *r* values as indicated in the lower half of the circle.

In the control group of 5 mice a total of 382 SWRs were detected during periods where the mouse was at rest. The waveform of the averaged raw SWR activity showed the characteristic combination of a negative deflection and high-frequency oscillations (Fig. 3a). Time-Frequency analyses of LFPs around the SWRs indicated that the detected SWRs had same peak frequency and duration as reported in literature (Fig. 3b) (for a recent review on SWR see ref. 29).

We used Rayleigh circular statistics to analyze the distribution of SWR events relative to the phases of the respiratory cycle during resting conditions, i.e. with respiratory frequencies not exceeding 4 Hz. This analysis revealed a significantly increased probability of SWR events during the early expiration phase of respiration (Rayleigh test: n = 382; r = 0.14; z = 7.35; p = 0.02). As illustrated in the polar plot in Fig. 3c, the respiratory phase during which SWR probability was highest (around 210°) corresponded to early expiration (Fig. 3c).

### Effect of inhibiting respiration-locked OB activity on respiration- locked CA1 activity

**Figure 4:**
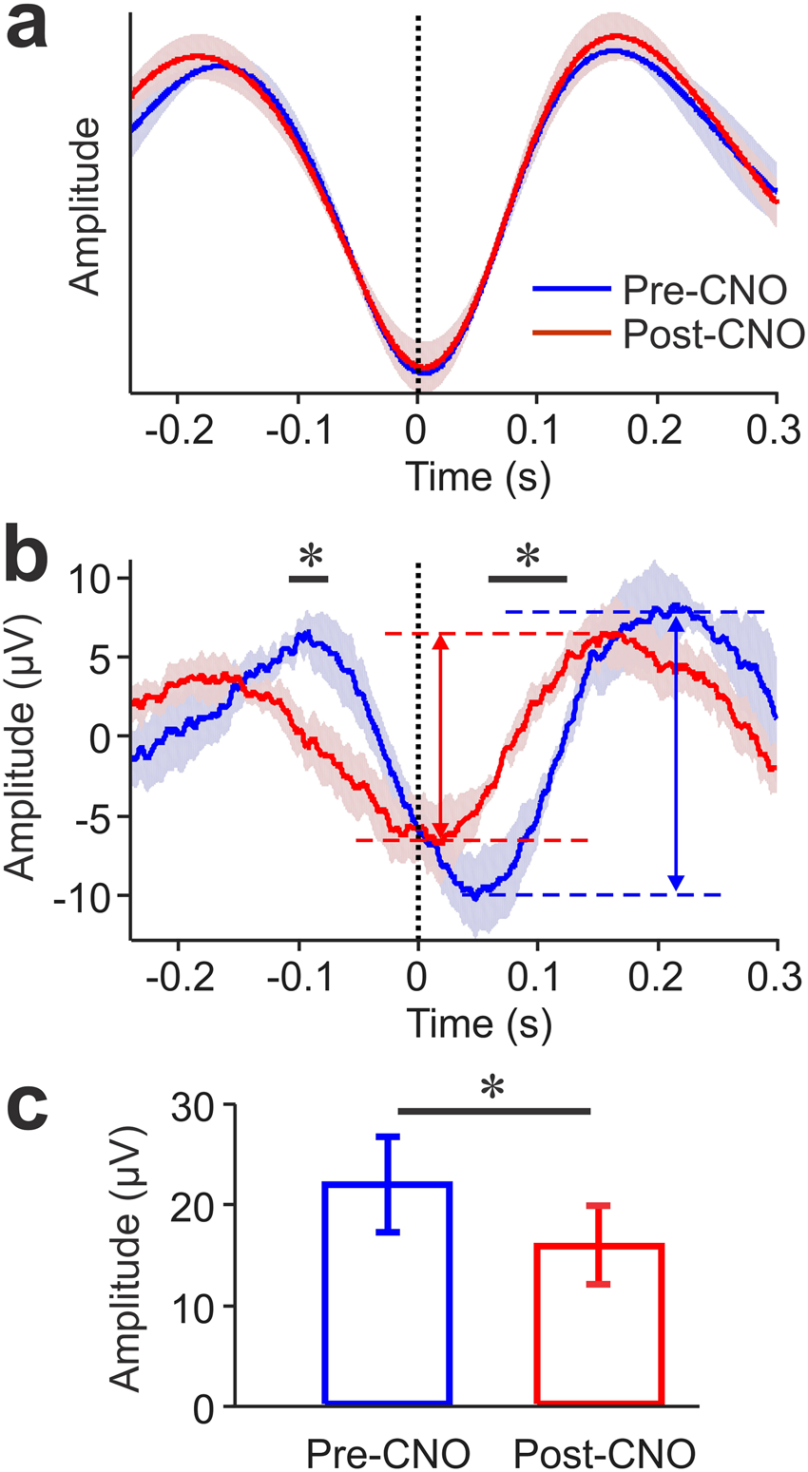
Changes in respiration and local field potential (LFP) recorded in hippocampal CA1 region following Clozapine N-oxide (CNO) injection in DREADD-mice (n = 4). (**a**) Group-average respiratory traces before (blue) and after (red) CNO-injection aligned on the end of expiration (t = 0). (**b**) Group-average LFP traces aligned on the end of expiration before and after CNO injection. Color code as in (**a**). Horizontal black bars mark times where the two traces differ significantly (two-Sample t-test: *p < 0.05). The amplitude of respiration-locked LFP oscillations was determined by measuring the voltage differences between LFP maxima and minima before and after CNO injection (red and blue dashed horizontal lines mark maximal and minimal voltage values in the corresponding average LFP traces). Blue and red vertical double-arrows indicate peak-trough voltage differences for pre and post CNO injection measurements, respectively. (**c**) The amplitude of average respiration-locked LFP oscillations in CA1 was significantly reduced when OB bulb activity was inhibited by CNO injections. Error bars represent standard error of the mean. (Paired-t-test: *p < 0.05).

Using 4 mice with OBs injected with DREADDs vector we investigated the effect of respiration-locked OB activity on hippocampal LFP oscillations. Hippocampal LFP activity contains slow-frequency oscillations in the delta and theta range that have been shown to be phase- locked to nasal respiration^11, 12^. We compared the amplitudes of respiration-aligned LFP activity in the hippocampus immediately before and 30-min after CNO-injection in OB-DREADD treated mice. Respiratory and LFP activity were aligned on the end of expiration. The respiratory waveform was not changed by CNO-injection (Fig. 4a). However, the respiration-aligned, average LFP signal shows a significant reduction in the amplitude of respiration-locked oscillations (Two-Sample t-test: p < 0.05). Figure 4b shows group mean LFP signals aligned on the end of expiration as measured before and after OB inhibition by CNO injection. Dashed horizontal lines in the panel illustrate how the amplitude of the respiration-locked oscillation in CA1 was determined by measuring the voltage differences between LFP minima and maxima (peak-to-trough voltage differences). Inhibiting OB bulb activity significantly reduced the amplitude of respiration-locked oscillations in CA1 (Fig. 4c).

### Effect of respiration-locked OB activity on SWR event timing

**Figure 5:**
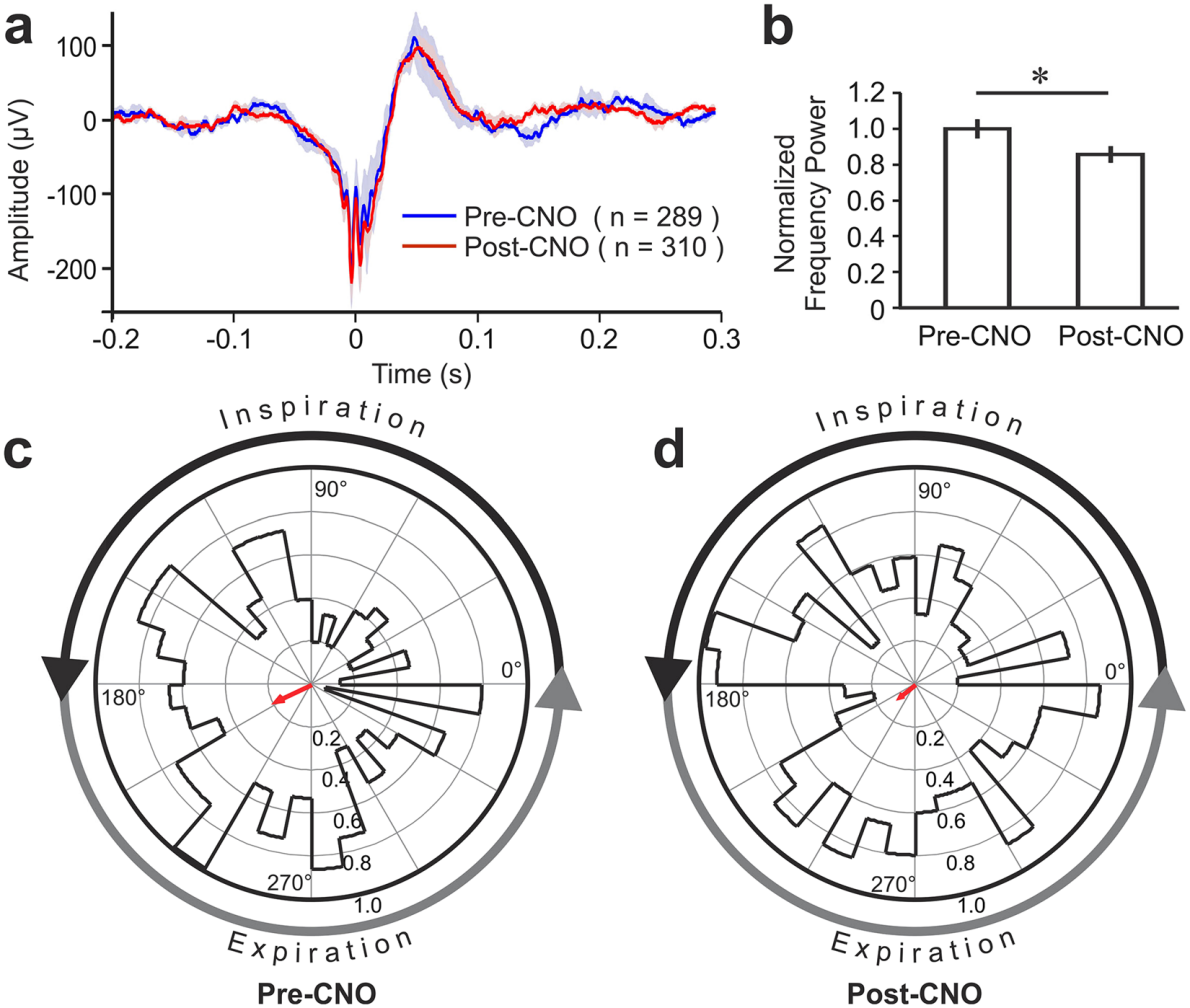
Changes in hippocampal sharp-wave ripple (SWR) activity before and after Clozapine N-oxide (CNO) Administration in mice with bilateral injections of DREADD vector in OB. (**a**) Average LFP waveform aligned on the SWR onset (t = 0). Solid blue and red lines represents mean LFP traces pre- and post-CNO treatment, respectively. Shading represents standard error. (**b**) Comparison of normalized spectral power within the 100–200 Hz frequency band pre- and post-CNO treatment. Two-Sample t-test: *p < 0.05. (**c**) Polar coordinates (°) for SWR activities during respiratory cycles, showing normalized vectors (thicker line areas) and mean vector length (red arrow; r) determined by circular statistics (Rayleigh test). 0° represents the end of expiration. The largest circle represents the maximum r (1.0). Pre-CNO: r = 0.18; z = 9.73; p < 0.001; Post-CNO: r = 0.09; z = 2.53; p = 0.278.

Finally, we asked whether the SWR activity was influenced by respiration-locked OB activity. In the four DREADD treated mice we compared the amplitudes, ripple frequency and the timing of SWR events relative to the phase of respiration before and after inhibition of respiration-locked OB activity by CNO injection. In all four mice combined we detected a total of 289 and 310 SWR events in comparable time periods before and 30 min after CNO injections, respectively. Inhibiting respiration-locked OB activity did not alter the amplitude of SWRs (Fig. 5a). However, the power of the ripple oscillation measured with the 100–200 Hz frequency range was significantly reduced following CNO-injection (Fig. 5b; Two-Sample t-test: p < 0.05). Analysis of SWR timing relative to the respiratory cycle prior to CNO injection revealed a significantly increased probability of SWR events during early expiration at a phase angle of around 210° (Fig. 5c; Rayleigh test: r = 0.18; z = 9.73; p = 0.001). This is consistent with results from the control group of 5 mice that did not receive DREADD treatment (Fig. 3c). Analysis of measurements taken 30 min after CNO-injection no longer showed a significant relationship between respiratory phase and SWR events (Fig. 5d; Rayleigh test: r = 0.09; z = 2.53; p = 0.278).

## Discussion

In this study we provide the first evidence that hippocampal SWR generation is modulated by respiration and that the influence of respiration on SWRs requires normal OB activity. Our evidence is based on experiments involving the simultaneous observation of hippocampal SWR activity and respiratory behavior in awake, head-fixed mice. We conducted our experiments under two main conditions: with the OB intact or with OB activity inhibited using neurochemical (DREADD) techniques. The main new findings are that the probability of the hippocampal network to generate SWR activity is significantly increased during the early phase of the expiration, and that this modulation of SWR generation depends on an intact OB. Taking into account the slow buildup of excitatory activity that has been shown to precede the high-frequency ripple^30^, the phase of increased probability would be slightly advanced to late inspiration.

We suggest that these findings are functionally relevant for memory formation and recall, as they tie in with recent results from a study in humans, which demonstrated a significant link between memory function and the phase of nasal respiration^27^. In this study subjects were shown pictures of real-world objects and were later asked to identify those objects out of a larger collection that contained an equal number of new objects. The probability to correctly identify previously seen objects was significantly higher for objects presented during the inspiration phase, both in the learning and the recall phase of the experiment^27^. Without knowing the timing of SWRs in this experiment relative to object presentation, recognition or response we cannot directly link the preferred phase of SWR timing in mice to the human results. However, the results from this study relate to our findings in two important ways: 1) Zelano *et al*. demonstrated that memory function is modulated by respiration on a cycle-by-cycle basis and 2) the influence of respiration on the reliability of memory recall was only observed during nasal respiration and not when subjects were breathing through the mouth^27^. Our results thus suggest a possible neuronal mechanism underlying the modulation of memory function with the phase of nasal respiration, as described by Zelano and colleagues^27^.

Our findings also raise the question of how respiration-locked OB activity can modulate the probability of SWR generation. Current data suggest that SWRs are intrinsically generated within the hippocampal network^31^, as they are also generated in hippocampal slice preparations^32, 33^. The probability of a SWR event in the intact brain could thus be modulated by respiration-locked activity reaching the hippocampus via the entorhinal cortex^11, 12^. As demonstrated here by us (Fig. 4) and previously by others^11, 12^, the effects of respiration-locked synaptic activity reaching the hippocampus via the entorhinal cortex can be directly observed in form of respiration-locked LFP oscillations. Our results show that loss of OB activity significantly reduces the amplitude of respiration-locked oscillations (Fig. 4). We hypothesize that the reduced modulation of hippocampal network excitability is no longer sufficient to cause SWR events generation to be phase-locked to respiration.

A second potential mechanism is less direct as it involves the suppression of SWR by cholinergic projections to the hippocampus from medial septal neurons^34^. Manns and colleagues reported that neurons in the basal forebrain nuclei, including cholinergic neurons, showed rhythmic spike activity that was highly correlated with respiration-locked OB activity^35^. Optogenetic activation of medial septal neurons, which provide cholinergic input to the hippocampus, has been shown to effectively suppress the generation of SWR in the hippocampal network^34^. A cholinergic input to the hippocampus that is modulated in phase with respiration could thus result in a rhythmic suppression of SWR and could create the respiration-locked bias we observed.

In summary, there is increasing recognition of the influence of the respiratory phase on various aspects of brain activity, including activity linked to cognitive functions^2, 8, 9, 27, 36^. Cognitive and emotional effects of breathing exercises consisting of practicing specific patterns of breathing have been the subject of several studies^37–45^. By contrast, the investigation of the influence of respiratory phase on brain function on a cycle-by-cycle basis is still in its infancy. The surprising discovery of the influence of respiratory phase on memory and memory-controlling neuronal activity is clearly relevant for understanding the fundamental mechanisms of memory. Since respiration is easily monitored and manipulated, these new findings may also prove to be of use in a clinical context, either as a diagnostic tool by linking abnormal respiratory patterns to memory deficits or for the treatment of memory deficits, e.g. by using biofeedback to identify respiratory patterns that enhance respiration-SWR phase locking.

## Methods

### Animals

Nine adult male mice (C57BL/6J) were used for investigation of hippocampal SWR activity, divided into a control group (n = 5) and a group that received olfactory bulb DREADD-treatment (n = 4). Four additional DREADD-treated mice were used to verify that CNO injections reliably inhibited OB activity. Mice were housed in a breeding colony at the University of Tennessee Health Science Center animal facilities with 12-hour light/dark cycles in standard cages with free access to food and water. All animal procedures were performed in accordance with the NIH Guide for the Care and Use of Laboratory Animals (2011). Experimental protocols were approved by the Institutional Animal Care and Use Committee.

### DREADD-transfection procedures in the OB

For controlled inhibition of respiration-driven OB activity, animals received bilateral 100 nL injections of AAV8-CamKIIa-HA-hM4D(Gi)-IRES-mCitrine (Addgene, Cambridge, MA) to the main OB (AP 4 mm, ML 0.85 mm, Depth 0.5 mm). Vectors were infused at a rate of 100 nL/min using a microsyringe (Neuros 1 uL, Hamilton Instruments, Reno, NV) and quintessential stereotaxic injector (Stoelting Instruments, Wood Dale, IL). The syringe needle was held stationary for 1 minute pre-injection and 2 minutes post-injection to allow for expansion of compressed tissue and diffusion of fluid into the tissue, respectively. Surgical sites were then covered with Kwik-sil epoxy (World Precision Instruments, Sarasota, FL) and the skin was closed with cyanoacrylate glue. Mice were given analgesic medication (0.05 ml carprofen solution, s.c.) and returned to their home cage for 4 weeks of viral incubation before preparation for recording.

### Clozapine-N-Oxide administration

Clozapine-N-Oxide (CNO) powder (Sigma-aldrich, St. Louis, MO) was dissolved in 5% DMSO and sterile injectable saline to create an ip-injectable solution. To initiate inhibition of OB activity, mice were injected with a dose of 5 mg/kg CNO in solution. Recordings were taken continuously over the next hour to observe electrophysiological changes as the drug took effect.

### Surgery

Mice were surgically prepared for awake, head-fixed, electrophysiological recordings. Surgical anesthesia was initiated by exposing mice to a mix of 3% isoflurane in oxygen in an incubation chamber. Anesthesia was maintained with 1–2% isoflurane in oxygen during surgery using an Ohio isoflurane vaporizer (Highland Medical Equipment, Deerfield, IL, USA). Rectal temperature was maintained at 37–38 °C with a servo-controlled heat blanket (FHC, Bowdoinham, ME, USA). To prepare for electrophysiological recordings from left CA1 region of hippocampus, a round skull opening (1.0–1.5 mm diameter) over the left hippocampus was made using a dental drill (Microtorque II, RAM Products, Inc., USA) without damaging the underlying dura (Fig. 1a; AP 2.3 mm; ML 2.0 mm). A cylindrical plastic recording chamber (4.5 mm diameter and 5 mm height) was placed over the skull openings and a metal head-post was mounted on the skull for head fixation during experiments. The chamber and head-post were embedded in acrylic cement and anchored to the skull bone using three small skull screws. The chamber was completely filled with triple antibiotic ointment. While still under anesthesia, mice were injected subcutaneously with Carprofen solution (0.05 ml; 50 mg/ml) to alleviate pain. The same surgical methods were used on 4 DREADD treated mice to implant thermistor probes above the nasal cavity to monitor respiratory activity^46^ and to prepare the mice for recordings from the OB (AP 4 mm, ML 0.85 mm). A postsurgical recovery period of 3–4 days was allowed before electrophysiological experiments.

### Electrophysiological experiments

Mice were adapted to the head-fixed position by placing them in the head holder for increasing amounts of time before the first recording session. Prior to each recording session, the chambers were cleaned and filled with sterile saline solution. Two extracellular recording electrodes (glass insulated tungsten/platinum; 80 μm diameter; impedance: 3.5–5.0 MΩ) were used to record LFPs. During experiments, the guiding tubes of a computer-controlled microdrive (Thomas Recording, Germany) were lowered into the saline-filled recording chamber to a distance of less than 1 mm from the dura surface. The stainless steel guiding tubes also served as reference electrodes and were electrically connected to the brain tissue via the saline solution. Two recording electrodes (350 μm apart) were slowly advanced through the intact dura into the neocortex directly overlying the hippocampal CA1 region and eventually into the CA1 proper for recordings. The accuracy of electrode tip positioning in CA1 was verified by histological examination of electrolytic lesions (10 μA; 12 s) created to mark the recording site (Fig. 1b). During the recordings, penetration depth and the appearance of characteristic SWRs in the LFP signal^29^ (Fig. 1c–e) were routinely used to verify electrode placement in CA1. All signals were band-pass filtered at 0.1 Hz–200 Hz, digitized at 2 kHz and saved to a hard-disk (CED 1401 and Spike2 software, Cambridge Electronic Design, U.K.). Respiration was simultaneously recorded using a thermistor that was positioned near a nostril^46^.

For mice in the control group, LFPs in the CA1 region were continuously recorded for at least 20 min. In DREADD-treated mice, LFP recordings were continued for a minimum of 50 min, including 10 min before and 40 min after CNO-administration.

### Data analysis

#### Detection of SWR activity in the CA1

For the analysis of hippocampal SWR, raw LFPs were band-pass filtered for the frequency range of SWR (150–200 Hz) (Fig. 1e). The mean amplitude and standard deviation of LFP amplitude fluctuation across each 1 min data block were calculated from the band-pass filtered signal. Potential SWR onsets were detected as LFP values larger or smaller than the average LFP value by ±3 SDs (Rothschild *et al*., 2017). A minimum of 5 ripple-frequency oscillation cycles (with the corresponding 5 consecutive voltage peaks exceeding 3 SD) were required to define a SWR. The time of the 3 SD threshold crossing of the first voltage peak of the oscillation defined the SWR onset. The end of a SWR was marked as the first LFP voltage that fell within the ± 3 SDs voltage range around the mean LFP, with the following voltage values remaining within this range for 200 ms. Based on this criterion, we treated SWRs separated by at least 200 ms as two distinct SWR events. SWR had to be detected on both recording electrodes.

It is relevant to mention that the high-frequency ripple component of the SWR complex is preceded by a slow build-up of excitatory activity^30^. In slice experiments this slow buildup has been shown to start between 50 and 60 ms before the onset of the high-frequency ripple^30^. There is no reliable way to detect the onset of the slow buildup in the LFP recorded *in vivo*, which is why we used the reliably detectable onset of the high-frequency ripple activity as the temporal align for our analyses.

#### Time-frequency analysis of LFP

To examine time-frequency aspects of SWR activity in the CA1 region of the hippocampus, LFPs were analyzed using an open-source software FieldTrip^47^. Sections of LFP-data from 0.2 s before and 0.2 s after SWR-onsets were selected for the performance of SWR-aligned Time-Frequency Analysis (FieldTrip function: ft_freqanalysis; Frequency-Rang: 100–200 Hz; Slide-Window: 0.2 s; Step: 1 ms).

#### Statistical analyses

Two-Sample t-test and Paired t-test were used to analyze changes in amplitude and power spectrum of SWR activities. Circular statistics (Rayleigh tests) were used to analyze SWR distribution during respiratory cycles. The respiration-entrained SWR activities were illustrated in polar plots of the distribution of SWR-timing as a function of respiratory phase (polar plots). The degree of entrainment was determined by circular statistics (Rayleigh test)^48^ and demonstrated by the mean vector length (r). Greater r indicated greater activity entrainment by the respiratory cycles. Moreover, the angular position of the mean vector in polar plots indicted the preferred phase for SWR activity and was related to the respiratory phase at which the most activity was distributed. In the present study, 0° in the polar plot represents the end of expiration as monitored by a thermistor placed in front of mouse nostrils^46^. Average spectral power was calculated within the 100 to 200 Hz frequency band and was normalized to the maximum value in each mouse. Unless specified otherwise, figures represent results as mean ± standard error.

### Verification of OB activity suppression by DREADD activation

For verification of DREADDs-induced disruption of respiration-locked neuronal oscillations in OB, respiratory activity was continuously monitored in 4 mice by a thermistor surgically implanted above the mouse nasal cavity^46^. Prior to CNO injection OB LFP oscillations were phase-locked to respiration (Fig. 2a). CNO injection eliminated the phase-locking of OB LFP oscillations to respiration (Fig. 2b). Waveform correlation analysis between the thermistor signal and OB LFP activity was used to quantify the relationship between the nasal airflow and OB activity. The peak cross-correlation values of the thermistor and LFP signals were calculated before and after DREADD activation by CNO injection. CNO treatment significantly reduced the correlation between respiration and OB LFP activity (Fig. 2c; paired t-test, p < 0.01).

### Histological evaluation of recording location

At the end of the experiments, animals were deeply anesthetized and intracardially perfused with 0.9% NaCl and followed by 4% paraformaldehyde solution. Brains (including OB) were removed and fixed in 4% paraformaldehyde solution for a minimum of 24 hours. The accuracy of electrode positioning was verified post-mortem for all animals by reference to surface maps of the location of cortical areas and hippocampus^49^. Fixed brains were sectioned at 60 μm and mounted onto slides. Light microscopy was used to verify the accurate position of the recording electrode tip in the CA1 region of the hippocampus (Fig. 1b).
